# Attitudes and educational needs of emergency doctors providing palliative and end-of-life care in Hong Kong: a cross-sectional analysis based on a self-report study

**DOI:** 10.1186/s12904-021-00742-1

**Published:** 2021-03-23

**Authors:** Kwun Hang Wong, Li Chuan Marc Yang, Kam Wing Raymond Woo, Oi Fung Wong, Wing Yan Kwong, Choi Fung Tse, Shing Kit Tommy Lam, Hing Man Ma, Chau Hung Albert Lit, Hiu Fai Ho, Yau Ngai Shih

**Affiliations:** 1 Accident and Emergency Department, North Lantau Hospital, 8 Chung Yan Road, Tung Chun, Lantau Hong Kong; 2 Fong Tam Yuen Leung Emergency Medicine Centre, The Chinese University of Hong Kong Medical Centre, 9 Chak Cheung Street, Shatin, New Territories Hong Kong; 3 Accident and Emergency Department, Queen Elizabeth Hospital, 30 Gascoigne Road, Kowloon, Hong Kong; 4 Department of Medicine and Geriatrics, Caritas Medical Centre, 111 Wing Hong Street, Sham Shui Po, Kowloon, Hong Kong; 5 Accident and Emergency Department, Tin Shui Wai Hospital, 11 Tin Tan Street, Tin Shui Wai, New Territories Hong Kong; 6 Accident and Emergency Department, Poh Oi Hospital, Au Tau, Yuen Long, New Territories Hong Kong; 7 Accident and Emergency Department, Princess Margaret Hospital, 2-10 Princess Margaret Hospital Road, Lai Chi Kok, Kowloon, Hong Kong

**Keywords:** Palliative care, End-of-life care, Accident and emergency department, Educational needs, Dying

## Abstract

**Background:**

Due to the ageing population in Hong Kong, the importance and need of palliative care and end-of-life (EOL) care are coming under the spotlight. The objectives of this study were to evaluate the attitudes of emergency doctors in providing palliative and EOL care in Hong Kong, and to investigate the educational needs of emergency doctors in these areas.

**Methods:**

A questionnaire was used to study the attitudes of ED doctors of six different hospitals in Hong Kong. The questionnaire recorded the attitudes of the doctors towards the role of palliative and EOL care in EDs, the specific obstacles faced, their comfort level and further educational needs in providing such care. The attitudes of emergency doctors of EDs with EOL care services were compared with those of EDs without such services.

**Results:**

In total, 145 emergency doctors completed the questionnaire, of which 60 respondents were from EDs with EOL care services. A significant number of participants recognized that the management of the dying process was essential in ED. Providing palliative and EOL care is also accepted as an important competence and responsibility, but the role and priority of palliative and EOL care in ED are uncertain. Lack of time and access to palliative care specialists/ teams were the major barriers. Doctors from EDs with EOL care services are more comfortable in providing such care and discuss it with patients and their relatives. Further educational needs were identified, including the management of physical complaints, communication skills, and EOL care ethics.

**Conclusions:**

The study identified obstacles in promoting palliative and EOL care in the EDs Hong Kong. With the combination of elements of routine ED practice and a basic palliative medicine skill set, it would promote the development of palliative and EOL care in Emergency Medicine in the future.

**Supplementary Information:**

The online version contains supplementary material available at 10.1186/s12904-021-00742-1.

## Background

An ageing population imposes significant burdens on different aspects of the society at large, particularly on the demands of the health care services. Hong Kong too is facing the same problem. The proportion of elderly people – aged 65 and above – is expected to increase from 16% in 2016 to 31% in 2046. In 2014, there were approximately 46,000 deaths in Hong Kong [1], of which about 80% were elderly people. Care for elderly patients with multiple comorbidities and patients with terminal and incurable diseases focuses on improving the quality of life, i.e. palliative and end-of-life (EOL) care, instead of aggressive disease-directed treatments. Palliative care is defined by the World Health Organization (WHO) as “an approach that improves the quality of life of patients and their families facing the problem associated with life-threatening illness, through the prevention and relief of suffering by means of early identification and impeccable assessment and treatment of pain and other problems, physical, psychosocial and spiritual” [2].

EOL care involves providing care to imminently dying patients and includes important elements of palliative care. According to the General Medical Council of the United Kingdom [3], patients are ‘approaching the end of life’ when they are likely to die within the next 12 months. This includes patients whose death is imminent (expected within a few hours or days) and also those with:

1. advanced, progressive, incurable conditions
2. general frailty and co-existing conditions that meant they are expected to die within 12 months
3. existing conditions if they are at risk of dying from a sudden acute crisis in their condition
4. life-threatening acute conditions caused by sudden catastrophic events

The use of healthcare services rises in the last 6 months, especially in the last 2 months prior to death. The average number of ED attendances and hospitalization days of elderly patients in their last year of life are 5 and 10 times of average, respectively [4]. Therefore, due to the combination of an aging population and increased burdens of chronic diseases, emergency doctors are expected to play a more crucial role in providing care for this group of patients, not limiting to acute emergency care but also good EOL care.

Emergency physicians have been traditionally tasked with many roles [5, 6]. With the above-mentioned differences in the characteristics of an ageing population, emergency physicians have already begun promoting EOL care in their practice. For example, the American Board of Emergency Medicine has established a subspecialty of hospice and palliative medicine in 2006 [7]. Research on implementing palliative and EOL care in EDs has been conducted in different countries. Most of the research subjects were emergency nurses [8–11] and only a few studies focused on emergency doctors [12–14]. EOL care is a potential field of development in Emergency Medicine (EM) in Hong Kong. However, research on the perceptions from the local emergency doctors on the provision of EOL care in the ED is still lacking.

### Objectives

The objectives of this study were to evaluate the attitudes of Hong Kong emergency doctors in providing palliative and EOL care, and to investigate the educational needs for emergency doctors in administering palliative and EOL care. In addition, the findings among emergency doctors of EDs with palliative and EOL care services were compared with those of EDs without such services.

## Methods

### Study design

This was a multicentre, cross-sectional questionnaire study. We collected data on the emergency doctors’ attitudes and perceived educational needs using a self-administered questionnaire. The returned questionnaires were then gathered and analysed together. The findings among emergency doctors of EDs with EOL care services were compared with those of EDs without such services. The reporting of this analysis is compliant with the Strengthening the Reporting of Observational Studies in Epidemiology (STROBE) Statement: guidelines for reporting observational studies [15] with modification suggested by the review of Bennett et al. [16].

### Setting

The participants were recruited from six EDs out of 18 hospitals of Hong Kong. Among them, North Lantau Hospital (NLTH) and Queen Elizabeth Hospital (QEH) provided ED-based EOL care services (see Table 1). In the QEH ED, EOL care was provided by a dedicated team of emergency physicians with an inpatient bed inside the Emergency Medicine Ward (EMW) [11, 17] Consultations from other inpatient departments were assessed by the on-duty emergency physicians and required terminally ill patients would be taken over to the EMW for treatment. Some patients would also be admitted to EOL care services directly from the resuscitation bays. In NLTH, terminally ill patients requiring EOL care were identified by the ED team and directly admitted to the EMW for treatment. Critically ill patients on whom life-sustaining treatment would prove futile were also included in the EOL care programme after consensus reached among the care team, patients and their families regarding the withholding or withdrawing of life-sustaining treatment [6]. EOL care services in both of these two units consisted of four aspects, namely physical symptoms control, management of psychological symptoms and relieve of psychological distress, spiritual support and relieve existential pain and social support. Palliative care specialists were not involved. The maximal length of stay of would be 2 days (QEH) or 7 days (NLTH); afterwards, the patients would be transferred to rehabilitation hospitals or other medical wards. There were no EOL care services in the EDs of the Tin Shui Wai Hospital, Pok Oi Hospital, Princess Margaret Hospital and Tuen Mun Hospital during the time of survey.

**Table 1:** Characteristics of participating hospitals

Hospital	Type of healthcare delivered	EOL service available in ED	Number of respondents vs total staff	ED attendance per year (2018–2019)	Number of EMW beds
North Lantau Hospital	Secondary	Yes	28/33	93,057	60
Queen Elizabeth Hospital	Tertiary	Yes	34/44	185,515	80
Princess Margaret Hospital	Tertiary	No	23/34	123,698	34
Tuen Mun Hospital	Tertiary	No	30/41	173,467	30
Pok Oi Hospital	Secondary	No	16/21	110,093	40
Tin Shui Wai Hospital	Secondary	No	16/22	109,642	32

Remarks:

*ED* Emergency Department, *EOL* End of Life, *EMW* Emergency Medicine Ward

### Participants

The target participants were the emergency doctors, including emergency medicine trainees, working in the EDs of the Queen Elizabeth Hospital, North Lantau Hospital, Tin Shui Wai Hospital, Pok Oi Hospital, Princess Margaret Hospital and Tuen Mun Hospital. Doctors not working regularly in the EDs, e.g. trainees rotated from other departments and part-time staff, were excluded.

### Variables

Baseline variables of each participant were collected in the questionnaire. Basic trainees of emergency medicine are fully licensed doctors who are undergoing specialist training but had not passed the intermediate examination of the Hong Kong College of Emergency Medicine. They typically had less than 3 years of experience. Higher trainees were fully licensed doctors who are undergoing specialist training that had passed the intermediate examination of the Hong Kong College of Emergency Medicine but not the final exit examination of the College, they typically had less than 6 years of training. Items and variables in the questionnaire are discussed separately.

### Data sources and measurement

#### Description of the research tool

The contents of the questionnaire were derived from a validated questionnaire [18], an overseas palliative and EOL care study [19] and local expert opinions. Permissions for using the content of the questionnaire and the overseas study had been obtained from the authors. The content of the questionnaire, apart from the recording the baseline variables of the participants (Part A, questions 1–9), assessed two main dimensions including their attitudes in providing palliative and EOL care {Part B, (questions 10–24), where Likert ratings were obtained against 15 statements}, and 11 items on educational needs were discussed (Part C, questions 25–35). The participants were asked about their overall attitude in providing palliative and EOL care in their respective EDs. The questions in Part B were divided into four categories: the role of palliative and EOL care (discussed in statements 1–3,6,7), specific obstacles faced by doctors while providing such care (discussed in statements 4,8,9,11–14), the comfort level while providing palliative and EOL care (discussed in statements 5 and 10) and the overall attitude towards palliative and EOL care (discussed in statement 15).

### Instrument reliability and validity

The content of the questionnaire was validated by Rivera et al. [19]. We sought opinion from a panel consisting of three experienced emergency physicians and a palliative medicine specialist, for organization and appropriateness. The questionnaire was then modified according to their comments. Modifications include the addition of the term “EOL care” in the statements 1,2, 4–8, 10–14 in Part B e.g. statement 1 “Palliative care is an important competence for an emergency medicine physician.” was changed to “Palliative and EOL care is an important competence for an emergency medicine physician.”; the addition of statement 15 (overall impression towards palliative and EOL care) for investigation of any correlations with specific obstacles (statements 4,8,9,11 to 14); and the change of direction of statements 4,8,9,11–14 (i.e. specific obstacles) for calculation of correlation coefficients e.g. Part B statement 4 “My workplace has protocols or services addressing palliative and EOL issues.” to “My workplace does not have protocols or services addressing palliative and EOL care issues.”;

### Scoring method

A 5-point Likert scale was employed for each question in Part B of the questionnaire, which included the options strongly agree, agree, neutral, disagree and strongly disagree. Further education need were assessed in Part C where the participants indicate which aspects of palliative and EOL care they would want further education.

### Method of questionnaire administration

Paper copies of the questionnaire were distributed by the investigators through direct, personal communication. No personal identifying data was collected to maintain anonymity and data access was restricted to the investigators only.

### Bias

Since the original questionnaire was not validated by the Chinese population, we rectified this by modifying the questionnaire, with expert input mentioned before.

### Study size

All the ED medical staff of the six hospitals were included in the study and determined the sample size.

### Method of sample selection

Target participants were identified according to the most updated staff list from their respective EDs.

### Quantitative variables

The staff was divided into groups according to five-year experience intervals, this choice is arbitrary. No other quantitative data needed modification or grouping.

### Statistical methods

The Statistical Package for Social Sciences (SPSS) version 20.0 for Windows was used for analysis. Mean, standard deviations, Spearman correlation coefficient, frequency of variables and percentage were used to classify data. Scores were assigned for each of the 5-point Likert scale options for each question (5 = strongly agree while 1 = strongly disagree) and mean and median scores of each question were calculated to reflect the level of agreement for each item. In those items related to the participants’ attitude in providing EOL care, the correlations between the level of their attitude in the individual items and their overall attitude in providing EOL care in the ED were calculated using Spearman’s correlations coefficient.

## Results

### Participants

In total, 145 participants returned questionnaires out of the total staff of 195 in the EDs of the 6 hospitals (response rate 74.3%). Sixty participants were from EDs with EOL service (EOL group) and 85 participants were from EDs without EOL service (Non-EOL group). Both EOL and Non-EOL group had similar response rates (77.9 and 72.0%, respectively).

### Descriptive data

Details were shown in Table 2. One hundred male and forty-five female doctors were recruited for the study, with similar ratios in both groups (male: female, 2.21:1 in EOL group, 2.27:1 in Non-EOL group). Emergency doctors with experience equal to or less than 5 years constituted the main part of the respondents. In the EOL group, more emergency doctors received on-the-job training about palliative and EOL care, compared Non-EOL group (48.3 and 31.8% respectively). A significant number of emergency doctors in both groups did not receive any training (EOL: 46.7%; Non-EOL: 67.1%). The self-ratings for knowledge of palliative and EOL care were similar in both groups (EOL vs Non-EOL group: no knowledge: 25% vs 22.4%; general knowledge: 71.7% vs 77.6%).

**Table 2:** Baseline characteristics of respondents

Characteristics	Overall (*n* = 145)	EOL group (*n* = 60)	Non-EOL group (*n* = 85)
	n (%)	n (%)	n (%)
Male Gender	69%	68.3%	69.4%
Years of experience in ED			
< 5	50 (34.5)	18 (30)	32 (37.6)
5–10	31 (21.4)	15 (25)	16 (18.8)
10–15	27 (18.6)	8 (13.3)	19 (22.4)
15–20	15 (10.3)	7 (11.7)	8 (9.4)
> 20	22 (15.2)	12 (20)	10 (11.8)
ED training status			
Service doctor	16 (11.0)	5 (8.3)	11 (12.9)
Basic trainee	38 (26.2)	16 (26.7)	22 (25.9)
Higher trainee	29 (20)	13 (21.7)	16 (18.8)
Specialist	62 (42.8)	26 (43.3)	36 (42.4)
Training status in palliative and EOL care			
Specialist training	0 (0)	0 (0)	0 (0)
Short courses or other formal training not leading to a specialist qualification	4 (2.8)	3 (5)	1 (1.2)
On job training only	56 (38.6)	29 (48.3)	27 (31.8)
No training	85 (58.6)	28 (46.7)	57 (67.1)
Self-rating of knowledge of palliative and EOL care			
No knowledge	34 (23.4)	15 (25)	19 (22.4)
General knowledge only	109 (75.2)	43 (71.7)	66 (77.6)
Professional/ extensive knowledge	2 (1.4)	2 (3.3)	0 (0)
Experience of caring terminal patients			
Yes	112 (77.2)	52 (86.7)	60 (70.6)
No	33 (22.8)	8 (13.3)	25 (29.4)
Confidence in caring terminal patients			
All of the time	1 (0.7)	0 (0)	1 (1.2)
Most of the time	29 (20)	19 (31.7)	10 (11.8)
Undecided	38 (26.2)	15 (25)	23 (27.1)
Somewhat	29 (20)	12 (20)	17 (20)
Not at all	15 (10.3)	6 (10)	9 (10.6)
Self-rating of support in caring for terminal patients			
Yes	26 (17.9)	20 (33.3)	6 (7.1)
No	59 (40.7)	24 (40)	35 (41.2)
Don’t know	27 (18.6)	8 (13.3)	19 (22.4)

Remarks:

*ED* Emergency Department, *EOL* end-of-life

### Outcome data

#### Attitude in providing EOL care

Results are illustrated in Table 3. For the role of palliative and EOL care (statements 1–3,6,7), most emergency medicine physicians disagreed with the statement “Emergency medicine physicians are trained to save lives and not to manage death” (statement 3).

**Table 3:** Attitude in providing EOL care (Questionnaire Part B). (1: strongly disagree, 2: disagree, 3: unsure/mixed, 4: agree, 5: strongly agree)

	Statements	Overall (mean, SD)	EOL group (mean, SD)	Non-EOL group (mean, SD)	*P*-value (Mann Whitney U test)
1	Palliative and EOL care is an important competence for an emergency medicine physician.	3.34, 0.966	3.62, 0.922	3.14, 0.953	0.004*
2	I have a clear idea of the role of palliative and EOL care in the emergency department.	2.77, 0.936	3.08, 0.944	2.54, 0.867	0.001*
3	Emergency medicine physicians are trained to save lives and not to manage death.	2.45, 1.034	2.07, 0.899	2.72, 1.042	< 0.001*
4	My workplace does not have protocols or services addressing palliative and EOL issues.	2.83, 1.190	1.95, 0.910	3.46, 0.946	< 0.001*
5	I feel comfortable providing palliative and EOL care in the emergency department.	2.75, 1.011	3.32, 0.911	2.35, 0.882	< 0.001*
6	Palliative and EOL care should not be the responsibility of the emergency physician.	2.49, 1.017	2.07, 0.907	2.79, 0.989	< 0.001*
7	Palliative and EOL care should have a lower priority in the busy emergency department.	2.92, 1.131	2.53, 1.127	3.20, 1.056	0.001*
8	There is lack of access to palliative and EOL care specialists/ teams in the emergency department.	3.70, 0.883	3.42, 1.013	3.91, 0.718	0.002*
9	Having no access to communication with palliative care physician affects my ability to provide EOL care in the emergency department.	3.53, 0.842	3.43, 0.767	3.60, 0.889	0.104
10	I have difficulty discussing palliative and EOL issues with patients and/or their families.	3.08, 0.965	2.87, 0.929	3.22, 0.968	0.033*
11	I cannot identify patients who may need palliative and EOL care in the emergency department.	2.64, 0.926	2.45, 0.891	2.78, 0.931	0.035*
12	My lack of training in palliative and EOL care affects my ability to provide this service.	3.46, 0.882	3.27, 0.972	3.59, 0.791	0.046*
13	Fear of lawsuits leads me away from offering palliative and EOL care to potential candidate.	2.88, 0.947	2.65, 0.880	3.05, 0.962	0.016*
14	I do not have sufficient time during my shift to provide palliative and EOL care in the emergency department.	3.73, 0.899	3.62, 1.010	3.81, 0.809	0.296
15	The emergency department is not the best place for EOL discussions	3.30, 1.015	2.93, 1.056	3.55, 0.906	0.001*

Remarks

The role of palliative and EOL care: statements 1–3,6,7

Specific obstacles faced by doctors while providing such care: statements 4,8,9,11–14

The comfort level while providing palliative and EOL care: statements 5 and 10

The overall attitude towards palliative and EOL care: statement 15

*ED* Emergency Department, *EOL* end-of-life, *SD* standard deviation

Asterisk*: *p* < 0.05

For the specific obstacles (statements 4,8,9,11–14), statement 14 (“I do not have sufficient time during my shift to provide palliative and EOL care in the emergency department.”) had the highest score (mean 3.73, SD 0.899), followed by statement 8 (“There is lack of access to palliative and EOL care specialists/ teams in the emergency department.”) (mean 3.70, SD 0.883).

For the comfort level with palliative and EOL care (statements 5 and 10), the results showed that the respondents were slightly uncomfortable in providing palliative and EOL care in EDs (statement 5, mean 2.75, SD 1.011) though there was no definite difficulty related to the discussion with patients and/or their families about palliative and EOL issues (statement 10, mean 3.08, SD 0.965).

For overall attitude, i.e. statement 15 (“The emergency department is not the best place for EOL discussions”), the mean score was close to 3 (mean 3.30, SD 1.015).

#### Further education needs

The results were demonstrated in Table 5. Most of the respondents wanted to learn more about pain assessment and management in palliative and EOL care (*n* = 100, 69.0%), while a smaller group of participants was interested to learn about the management of spiritual and cultural aspects (*n* = 45, 31.0%).

### Main results

A significant number of participants recognized that management of death is essential in EDs. Lack of time and access to palliative and EOL care specialists/ teams were the major barriers. Emergency doctors might be uncomfortable providing the care, but it is not obviously difficult for them to discuss it with patients and relatives. Further educational needs, apart from the management of physical complaints like pain management, include aspects of communication skills and EOL care ethics.

### Other analysis

We have carried out correlation analysis between obstacles and impression of staff. We have also carried out a subgroup analysis of doctors in the EOL and Non-EOL units.

### Correlation between specific obstacles and overall impression

In order to evaluate the impacts of specific obstacles faced during the administration of palliative and EOL care in ED, Spearman’s rank correlation coefficients (r) were calculated for each obstacle. In Table 4, all r’s had the same sign i.e. positive, which meant that the preset opposing statements very unlikely recorded high scores while the score of overall attitude was low in the same questionnaire sample. The value of r was highest for statement 13 (“Fear of lawsuits leads me away from offering palliative and EOL care to potential candidate.”, *r* = 0.334) while that of statement 12 (“My lack of training in palliative and EOL care affects my ability to provide this service.”) was the lowest (*r* = 0.043). These correlation coefficients showed that the medicolegal issues would likely discourage the implementation of palliative and EOL care in EDs, while lack of training in palliative and EOL care would not have significant negative effects. However, in general all r’s were less than 0.5, which meant these obstacles only have weak associations with the overall attitude. Most of the *p*-values (except question 9 and 12) were less than 0.05, which indicated the differences in corresponding r’s were statistically significant.

**Table 4:** Obstacles (statements 4, 8, 9, 11 to 14) correlated with overall impression (statement 15)

Statements number	Statement	Correlation coefficient	*P*-value (Spearman rank correlation)
4	“My workplace does not have protocols or services addressing palliative and EOL issues.”	0.219	0.008*
8	“There is lack of access to palliative and EOL care specialists/ teams in the emergency department.”	0.172	0.039*
9	“Having no access to communication with palliative care physician affects my ability to provide EOL care in the emergency department.”	0.115	0.168
11	“I cannot identify patients who may need palliative and EOL care in the emergency department.”	0.171	0.040*
12	“My lack of training in palliative and EOL care affects my ability to provide this service.”	0.043	0.607
13	“Fear of lawsuits leads me away from offering palliative and EOL care to potential candidate.”	0.334	< 0.001*
14	“I do not have sufficient time during my shift to provide palliative and EOL care in the emergency department.”	0.213	0.010*

Remarks

Asterisk*: *p* < 0.05

### Attitude in providing EOL care (Table 3)

With respect to the role of palliative and EOL care, emergency doctors in the EOL group, compared with those who worked in EDs without EOL services, were more likely to agree that palliative and EOL care is an important competence (statement 1) and understand the role of palliative and EOL care (statement 2). Besides, they were more likely to disagree about the different importance of saving lives versus managing death (statement 3), the exemption of palliative and EOL care (statement 6) and the lower priority of palliative and EOL care (statement 7) in EDs. *P*-values of all these five statements were less than 0.05, which were statistically significant. For affirmative statements, Statement 1 had the highest score in the EOL group (mean 3.62, SD 0.922). For the non-EOL group, only Statement 2 showed significant deviation from neutral (i.e. a score of 3), and its score tended to be against the clarity of the role of palliative and EOL care in EDs.

For the specific obstacles, Statement 14 had the highest score (mean 3.62, SD 1.010) while Statement 4 had the lowest score (mean 1.95, SD 0.910), i.e. more emergency doctors in the EOL group thought they did not have enough time during their shifts to provide palliative and EOL care, despite confirmed presence of palliative and EOL care services in their EDs. In the non-EOL group, Statement 8 had the highest scores (mean 3.91, SD 0.718), while Statement 11 scored the lowest point (mean 2.78, SD 0.931). It indicated that the lack of access to palliative and EOL care specialists/ teams in the emergency department was the most salient concern, while the identification of patients in need of palliative and EOL care was not difficult for the emergency doctors in the non-EOL group. Most of the statements had *p*-values less than 0.05, except statements 9 and 14 (mean 0.104, 0.296 respectively).

With respect to the comfort level of administering palliative and EOL care, the emergency doctors in the EOL group felt more comfortable in providing such care and found it less difficult in discussing palliative and EOL issues with patients and/ or their relatives. The difference in the mean scores of the two groups was statistically significant (*p*-values of both statements were less than 0.05).

With respect to an overall attitude i.e. statement 15, the score of the emergency doctors in the EOL group was close to 3 (mean 2.93, SD 1.056) while non-EOL group’s mean score was 3.55 (SD 0.906), and it was statistically significant (*P*-value 0.001).

### Further education needs

In the EOL group, education regarding communication skills seemed to be most necessary (*n* = 42, 70%), while in the non-EOL group participants were more concerned about pain assessment and management (*n* = 60, 70.6%). In contrast, the educational needs for last offices and ritual arrangements were less emphasized in the EOL group (*n* = 24, 40%), while the emergency doctors in the non-EOL group were less interested in the management of spiritual and cultural aspects (*n* = 16, 18.8%).

For between-group (EOL and Non-EOL groups) comparison, only 4 of 11 statements had *p*-values less than 0.05 (“Management of death rattle”, “Management of feeding in EOL care”, “Management of psycho-social aspects of EOL care” and “Management of spiritual and cultural aspects in EOL”). There was a significant proportion of emergency doctors in the non-EOL group (above 60%) who did not indicate further need for education on these aspects, while about half of those in the EOL group expressed their need for further education on these aspects.

## Discussions

### Key results

As a city with the one of the densest populations, Hong Kong’s public hospitals are always crowded with patients. Therefore, the time limit in each shift would be a major barrier in providing palliative and EOL care in EDs and it is shown in the study (Table 3). However, the correlation with overall attitude is relatively weak (*r* = 0.213, *p* = 0.010). On the other hand, the overall mean score of Statement 7 of Part B of the questionnaire (“Palliative and EOL care should have a lower priority in the busy emergency department”) was close to neutral, with the second greatest value of SD. The EOL group had a lower mean score, i.e. tended to oppose the lower priority of palliative and EOL care in ED. It is reasonable that the busy working environment would discourage doctors to practice palliative and EOL care in EDs which is also suggested in other overseas studies [20, 21]. However, there is a significant proportion of staff who would like to practice palliative and EOL care in EDs. Combining the results of these two statements, we predict that if resources for palliative and EOL care services were available, emergency doctors would be more willing to provide such care.

Access to palliative and EOL care specialists or teams would affect emergency doctors’ desire to provide such care in EDs. The Spearman correlation coefficient of Statement 8 of Part B (“There is lack of access to palliative and EOL care specialists/ teams in the emergency department”) was 0.172, which was statistically significant. Providing round-the-clock backup in the form of palliative care physicians would lessen the barrier for palliative care in EDs [22, 23]. It may be possible to establish a palliative care liaison service modelled after the psychiatric liaison service to better support the emergency physicians.

Medicolegal issues have caused fear in every medical personnel. It was predictable that palliative and EOL care, as a new aspect in ED service, would induce stress in and among emergency physicians. The correlation between the fear of lawsuits with the overall attitudes towards palliative and EOL care was the highest among all specific obstacles {statement 13 (“Fear of lawsuits leads me away from offering palliative and EOL care to potential candidate.”; *r* = 0.334)}. Although it was statistically significant, it was not strong. Besides, its overall mean scores were close to neutral, with lower value representing the EOL group. The statistically significant difference between the two groups could be attributed to the availability of established protocols in EDs with EOL care services and clinical experience of palliative and EOL care in EOL group.

Emergency physicians managed various types of pain-related complaints. Even so, significant proportions in both groups (EOL - 66.7%; non-EOL - 70.6%; overall 69.0%) sought further education about pain assessment and management. Similarly, one of the distinct themes related to palliative care shown in the study of Smith et al. was inadequate training in pain management [24].

Emergency physicians are expected to formulate a plan of care and inform patients and relatives about the prognosis, goal of care, and the dying process in an organized manner within a short period of time. Marck et al. [12] demonstrated several difficulties faced by the Emergency staff when dealing with patients and their relatives while providing palliative and EOL care services in EDs for cancer patients. The difficulties included difference in expectations (prognosis, usefulness of further investigations and treatments), challenges for the staff (fear of liability or legal issues or criticism) and challenges related to systemic issues (limited information about the patients, limited time for assessment, lack of family presence and/or private room, perception of ED’s role as provider of active treatments). Therefore, further education about communication skills and EOL care ethics require much attention, as established in our study (refer to Table 5).

**Table 5:** Items indicated for further education needs (Questionnaire Part C)

Items	Overall (*n* = 145)	EOL group (*n* = 60)	Non-EOL group (*n* = 85)	*P* value (Chi square test)
	n (%)	n (%)	n (%)	
Pain assessment and management	100 (69.0)	40 (66.7)	60 (70.6)	0.615
Management of terminal delirium	82 (56.6)	39 (65)	43 (50.6)	0.085
Management of dyspnea	95 (65.5)	40 (66.7)	55 (64.7)	0.807
Management of death rattle	63 (43.4)	32 (53.3)	31 (36.5)	0.044*
Management of feeding in EOL care	62 (42.8)	34 (56.7)	28 (32.9)	0.004*
Management of psych-social aspect of EOL	62 (42.8)	35 (58.3)	27 (31.8)	0.001*
Management of spirituality and cultural aspects in EOL	45 (31.0)	29 (48.3)	16 (18.8)	< 0.001*
Bereavement management	78 (53.8)	34 (56.7)	44 (51.8)	0.560
Last office and ritual arrangement	47 (32.4)	24 (40)	23 (27.1)	0.101
Communication skills	92 (63.4)	42 (70)	50 (58.8)	0.169
EOL care ethics	97 (66.9)	39 (65)	58 (68.2)	0.683

Remarks

*EOL* end-of-life

Asterisk*: *p* < 0.05

### Limitations

There were several limitations for our analysis in addition to the usual limitation associated by survey studies (e.g. sampling bias, demand characteristic bias, desirability bias and non-response bias).

First, not all EDs of Hong Kong hospitals were included in the study. There were 534 Emergency physicians working in the EDs in public hospitals. Therefore, in this study we only sampled around 27% physicians working in public hospitals.

Second, the response rate for this study (74.3%) was quite satisfactory, compared to the usual response rate in surveys in the medical field [25–27]. However, the remaining portion of non-respondents may systematically differ in their responses, and the effect of this on the analysis is unknown.

Third, the questionnaire used in this study was modified without further tests on the validity and reliability, so the results might be biased. The questionnaire needed to be validated in the future should it be used again.

Fourth, palliative and EOL care could be divided into general and specialized aspects. We intended to focus on the attitudes towards the general aspect of such care and the relevant educational needs, but this intent was not clearly stated in the questionnaire. Therefore, the respondents’ choices would be affected.

Fifth, we do not have objective measurement of the actual knowledge of the participants and only relied on self-reported knowledge.

## Conclusion

To the best of our knowledge, this study was the first of its kind in Hong Kong about the attitudes of emergency physicians towards palliative and EOL care in ED. The results showed several obstacles during the implementation of palliative and EOL care in EDs. Further education, especially communication skills and ethical issues are recognized as necessary. Territory-wide studies would be useful to further clarify the issues and contribute to the development in this previously overlooked area in our profession. With the combination of elements of routine ED practice and a basic palliative medicine skill set, it would promote the development of Emergency Medicine in the future [28].

### Generalisability

Our current study is applicable to any urban emergency department planning to establish an EOL care service.

## Supplementary Information

**Additional file 1.**

**Additional file 2.**

## Data Availability

The datasets generated and analysed during the current study are available in the Mendeley Data repository [29], 10.17632/xdcrdjs48f.

## Abbreviations

ED: Emergency Department
EM: Emergency Medicine
EMW: Emergency Medicine Ward
EOL: End-of-Life
NLTH: North Lantau Hospital
QEH: Queen Elizabeth Hospital
SD: Standard Deviation
