# Supplementary material for: Attitudes and educational needs of emergency doctors providing palliative and end-of-life care in Hong Kong: a cross-sectional analysis based on a self-report study
Source: BMC Palliat Care. 2021 Mar 23;20:48. doi: 10.1186/s12904-021-00742-1 (PMC7988912; doi:10.1186/s12904-021-00742-1)
Supplement: Supplementary file 1 — Additional file 1. [file 12904_2021_742_MOESM1_ESM.docx]

**Questionnaire Version 7.0, Version Date 31/08/2019**

**Topic**: Attitude of emergency doctors in providing palliative and end-of-life (EOL) care in Hong Kong and education needs: a self-reported questionnaire survey

Part A: About you

Q1,3-9: Please indicate your answers by ticking the relevant box below.

1. Your gender

| Male |  |
| --- | --- |
| Female |  |

2. Your years of Accident and Emergency (A&E) experience (please put a tick in the correct box):

| 0-5 years |  | 10-15 years |  | More than 20 years |  |
| --- | --- | --- | --- | --- | --- |
| 5-10 years |  | 15-20 years |  |  |  |

3. Your A&E training status (please put a tick in the correct box)

| Service doctor |  |
| --- | --- |
| Basic trainee |  |
| Higher trainee |  |
| Specialist |  |

4. Your palliative care/ EOL care training (please put a tick in the correct box)

| Specialist training |  |
| --- | --- |
| Short courses or other formal training not leading to a specialist qualification e.g. Postgraduate Diploma in End-of-Life Care, The Chinese University of Hong Kong (PgD EOLC (CUHK)) |  |
| On job training only |  |
| No training |  |

5. Did your emergency department currently provide palliative and EOL care service? (Please put a tick in the correct box)

| Yes |  |
| --- | --- |
| No |  |

6. How would you rate your knowledge of palliative and EOL care?

| No knowledge |  |
| --- | --- |
| General knowledge only |  |
| Professional / extensive knowledge |  |

7. Have you ever looked after someone who was dying?

| Yes |  |
| --- | --- |
| No |  |

8. If you answered “yes” for question 7, how confident would you feel when looking after the person who was dying?

| Confident all of the time |  |
| --- | --- |
| Confident most of the time |  |
| Undecided |  |
| Somewhat confident |  |
| Not at all confident |  |

9. Did you feel that you had enough support to undertake this role?

| Yes |  |
| --- | --- |
| No |  |
| Don't know |  |

Part B: Attitude in providing EOL care

Please indicate how much you agree or disagree with each of the following statements, by ticking the box that best describes how you feel.

1 = strongly disagree, 2 = disagree, 3 = unsure/mixed, 4 = agree, 5 = strongly agree

| No | Statements | 1 | 2 | 3 | 4 | 5 |
| --- | --- | --- | --- | --- | --- | --- |
| 1 | Palliative and EOL care is an important competence for an emergency medicine physician. |  |  |  |  |  |
| 2 | I have a clear idea of the role of palliative and EOL care in the emergency department. |  |  |  |  |  |
| 3 | Emergency medicine physicians are trained to save lives and not to manage death. |  |  |  |  |  |
| 4 | My workplace has protocols or services addressing palliative and EOL issues. |  |  |  |  |  |
| 5 | I feel comfortable providing palliative and EOL care in the emergency department. |  |  |  |  |  |
| 6 | Palliative and EOL care should not be the responsibility of the emergency physician. |  |  |  |  |  |
| 7 | Palliative and EOL care should have a lower priority in the busy emergency department. |  |  |  |  |  |
| 8 | There is lack of access to palliative and EOL care specialists/ teams in the emergency department. |  |  |  |  |  |
| 9 | Having no access to communication with palliative care physician affects my ability to provide EOL care in the emergency department. |  |  |  |  |  |
| 10 | I have difficulty discussing palliative and EOL issues with patients and/or their families. |  |  |  |  |  |
| 11 | I cannot identify patients who may need palliative and EOL care in the emergency department. |  |  |  |  |  |
| 12 | My lack of training in palliative and EOL care affects my ability to provide this service. |  |  |  |  |  |
| 13 | Fear of lawsuits leads me away from offering palliative and EOL care to potential candidate. |  |  |  |  |  |
| 14 | I have sufficient time during my shift to provide palliative and EOL care in the emergency department. |  |  |  |  |  |
| 15 | The emergency department is not the best place for EOL discussions |  |  |  |  |  |

Part C: Further Education Needs

Please tick the boxes to indicate whether you would like future education on any of the

following topics:

| Pain assessment and management |  |
| --- | --- |
| Management of terminal delirium |  |
| Management of terminal dyspnea |  |
| Management of death rattle |  |
| Management of feeding in EOL care |  |
| Management of psycho-social aspect of EOL |  |
| Management of spirituality and cultural aspects in EOL |  |
| Bereavement management |  |
| Last office and ritual arrangement |  |
| Communication skills – breaking bad news, and discussion on prognosis and initiation of EOL care |  |
| EOL care ethics: Limitation of life-support therapy, DNACPR decision, advance directives and share-decision making |  |

This is the end of the survey. Thank you for your time.

Other comments:
