# Supplementary material for: Attitudes and educational needs of emergency doctors providing palliative and end-of-life care in Hong Kong: a cross-sectional analysis based on a self-report study
Source: BMC Palliat Care. 2021 Mar 23;20:48. doi: 10.1186/s12904-021-00742-1 (PMC7988912; doi:10.1186/s12904-021-00742-1)
Supplement: Supplementary file 2 — Additional file 2. [file 12904_2021_742_MOESM2_ESM.doc]

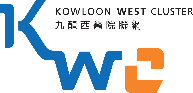

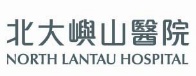

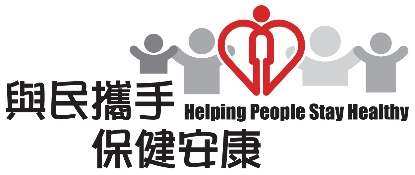


North Lantau Hospital

8 Chung Yan Road

Tung Chung, Lantau, N.T.

Tel: (852) 3467 7000

Fax: (852) 3467 7004

**北大嶼山醫院**

**新界大嶼山東涌松仁路8號**

**電話: (852) 3467 7000**

**傳真: (852) 3467 7004**


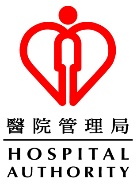


**Information Sheet**

**Attitude of emergency doctors in providing palliative and end-of-life care in Hong Kong and education needs: a self-reported questionnaire survey**

You are invited to participate in this research. Before you decide, it is important that you understand why the research is done and how you will be involved. Please read the information carefully. Ask if there is anything unclear or if you wish to obtain more information. Take time to decide whether you wish to participate in the research.

**Purpose of research**

Study Background

Aging population is a hot topic in the world, especially in the developed countries, because of the increasing burdens in different aspects in the society, including medical care. With the advance in medicine, elderlies with multiple comorbidities and patients with incurable diseases, such as organ failure and malignancies, could have a much longer life expectancy. With this background, specialty of palliative medicine was developed. Palliative medicine is defined by World Health Organization (WHO) as “an approach that improves the quality of life of patients and their families facing the problem associated with life-threatening illness and terminal disease, through the prevention and relief of suffering by means of early identification and impeccable assessment and treatment of pain and other problems, physical, psychosocial and spiritual problems” (1). Palliative medicine specialists are trained to provide specialized palliative care for patients with terminal illness and incurable disease. The American Academy of Hospice and Palliative Medicine defined palliative care as both a philosophy of care and an organized and highly structured system for delivering care with goals of preventing and relieving suffering, and supporting the best possible quality of life for the patients and their families, regardless of the stage of the disease or the need for other therapies. Palliative care extends the traditional disease-model of medical treatments to the goals of optimizing the function of the patient, enhancing the quality of life and facilitating own decision-making for the patient and the family. Palliative care is not only limited to the terminal stage of disease. On the other hand, it can be delivered concurrently with life-prolonging care or as the main focus of care (2). Palliative care is also not limited just to cancer conditions. Other incurable conditions also require a good palliative care. Most these incurable medical conditions are common medical illnesses encountered in the acute medical ward setting, including advanced cardiac failure, advanced chronic obstructive lung disease (COAD), chronic renal failure, dementia and other degenerative neurological diseases. However, the importance of palliative care for these incurable medical conditions is often overlooked.

The importance and need of palliative care is increasing all over the world (3). Hong Kong is also facing the problem of aging population. According to the data from the Census and Statistics Department, the proportion of elderly people aged 65 and above will increase from 16% in 2016 to 31% in 2046. The overall Hong Kong population will increase from 7.3 million to 8.2 million during the same period (4). In 2014 there were approximately 46 000 deaths in Hong Kong (5), in which about 80% were elderly people aged 65 or above. In addition, three forth of elderly people suffered from one or more chronic conditions according to the data from the Census and Statistics Department the in the same year (6). The demand of palliative care service rises with increasing number of elderly patients with multiple comorbidities. Despite the great demand of palliative care service, there are only 21 registered palliative medicine specialists in Hong Kong up till 2018. The lack of professional providers in palliative care leads to a service gap in nowadays health care system. The gap is anticipated to become larger in the future.

End-of-life (EOL) care, which is an important element in palliative care, encompasses the provision of care to the imminently dying patients. According to General Medical Council of the United Kingdom (7), patients are ‘approaching the end of life’ when they are likely to die within the next 12 months. This includes patients whose death is imminent (expected within a few hours or days) and those with:

1. advanced, progressive, incurable conditions
2. general frailty and co-existing conditions that mean they are expected to die within 12 months
3. existing conditions if they are at risk of dying from a sudden acute crisis in their condition
4. life-threatening acute conditions caused by sudden catastrophic events.

This guidance also applies to those extremely premature neonates whose prospects for survival are known to be very poor, and to patients who are diagnosed as being in a persistent vegetative state, for whom a decision to withdraw treatment may lead to their death.

In general, EOL care is a multidisciplinary approach care. It combines a broad set of health and community services including physicians, nurses, allied health professionals, medical social workers and chaplaincy. Provision of EOL care is not limited to the palliative medicine specialists. Physicians from other specialties, with proper training, are also eligible to provide good EOL care for the imminently dying patients. This helps fill up part of the service gap of palliative care in the healthcare system. The use of healthcare service rose in the last 6 months, especially in the last 2 months, of life. The average number of Accident and Emergency Department (AED) attendances and hospitalization days of elderly patients in their last year of life were 5 and 10 times of other elderly patients respectively. Therefore, with the combination of aging population and increased burden from chronic diseases, emergency doctors are expected to provide care to this group of patients more frequently and play more important role in providing EOL care.

Being a specialty focusing on the management of acute and emergency conditions, emergency medicine and active management are almost always put together traditionally. In Hong Kong, most AEDs don’t put much emphasis on EOL care. But with the abovementioned changes of population characteristics, some overseas emergency physicians have already started to promote EOL care in their AEDs. For example, the American Board of Emergency Medicine established a subspecialty of hospice and palliative medicine in 2006. (8) Researches on implementing EOL care in AED have been conducted in different countries. Most of the research subjects were emergency nurses (9-12) and a few studies focused on emergency doctors. (13-15) To conclude, emergency doctors and nurses were confident in symptom management, but not in the aspects of EOL care communication and related ethical issues. Major obstacles to provide EOL care in AED were inadequate support and training.

The AED of Queen Elizabeth Hospital (QEH) established the first comprehensive EOL care service in the Emergency Medicine Ward (EMW) in 2010. A qualitative study for the perception of nurses in the AED of QEH showed positive result of their EOL care service from the nursing staff perspective. The emergency nurses perceived that EOL care not only enhanced the patients’ last moment of life and facilitated the grief process of their relatives, but also enriched the professional development for the nursing staff. (12) A comprehensive EOL care program was also initiated in the EMW of North Lantau Hospital (NLTH) in 2017 upon the unique service need of the community. Similar to the EOL care service in QEH AED, the program in NLTH has become mature with protocol launched. EOL care is a potential field of development in the EM specialty in Hong Kong. However, research on the perceptions from the local emergency doctors on the provision of EOL care in the AED is still lacking.

Aim of the Study

1. To evaluate the attitude and the education needs of emergency doctors in providing EOL care in Hong Kong
2. To investigate the education needs for emergency doctors in EOL care

Duration of the Study

17^th^ December 2018 to 31^st^ December 2020

**Why are you chosen?**

A target number of 100 emergency doctors will be invited to volunteer as study subjects at their convenience. No previous study has been done on this subject.

**Do I have to participate?**

It is up to you to decide whether to participate or not. If you decide to participate, you will keep this information sheet. You will be free to withdraw at any time and without giving a reason.

**What happens if I participate?**

Participants will be invited to complete the questionnaire with an estimated duration of 15 minutes once at their convenience. If you would like to participate in the study, please return the completed questionnaire to Collection box no. 001 in NLTH AED in the sealed envelope provided on or before 31/5/2019.

Study Procedure

One piece of hard copy of the questionnaire will be given to the participant. The procedure is completed when the hard copy is returned to the investigators.

Outcomes of the Study

1. To evaluate the attitude and the education needs of emergency doctors in providing EOL care in Hong Kong

2. To investigate the education needs for emergency doctors in EOL care

**What are the benefits?**

The participants will be asked to be involved in the study on a voluntary basis at their convenience. No pro-rated payment will be provided for participating in the study. Being the participants of this study, they could contribute to the development of the palliative and end-of-life care in the Accident and Emergency Department, so they and other Emergency physicians can gain more knowledge and skills about this aspect and be more well prepared in the future in terms of daily practice and career advancement.

**What are the risks and disadvantages of participation?**

This study is questionnaire study and it does not involve real patients. Reasonable foreseeable risk will be minimal.

**What happens if new information is available?**

The researchers will inform you if new information about the questionnaire is available. You may decide whether to continue to participate in the research or not. If you decide to continue, you will be asked to send the questionnaire to the investigators again. If you choose to withdraw, you will be free to withdraw at any time and without giving a reason. On the other hand, the researchers may suspend your participation in the research should it be in your best interest.

**What happens if something goes wrong?**

If you are harmed by participating in this research, this is no special compensation arrangement.

**Will my participation be kept confidential?**

All information about you collected during the research will be kept strictly confidential. The data access will be restricted to authorized hospital staff and only in hospital premises. Data files will be password protected and only authorized personnel will have access to these files

**What about the research results?**

The research results will be written up as project dissertation and paper for journal publication on completion of the study. The subject cannot be identified in the report or publication.

**Who finances and organizes the research?**

The researchers receive no sponsor or funding for this research.

**Who has reviewed the research?**

The study has been approved by KWC REC (Kowloon West Cluster Research Ethics Committee).

**Inquiry contact**

Contact Person: Dr Wong Kwun Hang

Contact Number: 65709407

Email: wkh001@ha.org.hk

For enquiry on subject rights, please contact the KWC REC at 29901017 via telephone.

**Thank you for participating in the research. You will be given a copy of the information sheet.**

**References**

1. World Health Organisation (WHO). WHO Definition of Palliative Care. Available at: www.who.int/cancer/palliative/definition/en
2. American Academy of Hospice and Palliative Medicine. Position statements. Available at http://www.aahpm.org/positions/quality.html (accessed July 16, 2018).
3. Strengthening of palliative care as a component of comprehensive care throughout the life course. http://apps.who.int/gb/ebwha/pdf_files/WHA67/A67_R19-en.pdf?ua=1&ua=1 (accessed March 14, 2018).
4. Census and Statistics Department, Hong Kong Special Administrative Region. 2016 Population By-census and Hong Kong Population Projections 2015-2064.
5. Immigration Department and Hospital Authority, Hong Kong Special Administrative Region.
6. Census and Statistics Department, Hong Kong Special Administrative Region. (2015). Thematic Household Survey Report No. 58.
7. Treatment and care towards the end of life: good practice in decision making. Available at: https://www.gmc-uk.org/End_of_life.pdf_32486688.pdf (2010, accessed March 26, 2018).
8. Todd KH. Practically speaking: Emergency medicine and the palliative care movement. *Emergency Medicine Australasia*; 24: 4–6.
9. Beckstrand RL, Wood RD, Callister LC, et al. Emergency Nurses Suggestions for Improving End-of-Life Care Obstacles. *Journal of Emergency Nursing* 2012; 38.
10. Wolf LA, Delao AM, Perhats C, et al. Exploring the Management of Death: Emergency Nurses’ Perceptions of Challenges and Facilitators in the Provision of End-of-Life Care in the Emergency Department. *Journal of Emergency Nursing* 2015; 41.
11. Decker K, Lee S, Morphet J. The experiences of emergency nurses in providing end-of-life care to patients in the emergency department. *Australasian Emergency Nursing Journal* 2015; 18: 68–74.
12. Tse JWK, Hung MSY, Pang SMC. Emergency Nurses’ Perceptions of Providing End-of-Life Care in a Hong Kong Emergency Department: A Qualitative Study. *Journal of Emergency Nursing* 2016; 42: 224–232.
13. Marck CH, Weil J, Lane H, et al. Care of the dying cancer patient in the emergency department: findings from a National survey of Australian emergency department clinicians. *Internal Medicine Journal* 2014; 44: 362–368.
14. Shearer FM, Rogers IR, Monterosso L, et al. Understanding emergency department staff needs and perceptions in the provision of palliative care. *Emergency Medicine Australasia* 2014; 26: 249–255.
15. Russ A, Mountain D, Rogers IR, et al. Staff perceptions of palliative care in a public Australian, metropolitan emergency department. *Emergency Medicine Australasia* 2015; 27: 287–294.
